# Exosomal proteomic signatures correlate with drug resistance and carboplatin treatment outcome in a spontaneous model of canine osteosarcoma

**DOI:** 10.1186/s12935-021-01943-7

**Published:** 2021-05-01

**Authors:** Marcus A. Weinman, Stephen A. Ramsey, Haley J. Leeper, Jacqueline V. Brady, Andrew Schlueter, Stanislau Stanisheuski, Claudia S. Maier, Tasha Miller, Carl E. Ruby, Shay Bracha

**Affiliations:** 1 University of Vermont, Burlington, USA; 2 Oregon State University, Corvallis, USA; 3 Veterinary Diagnostics and Preclinical Research Services, Davis, USA; 4 Washington State University, Pullman, USA; 5 Texas A&M University, College Station, USA

**Keywords:** Osteosarcoma, Exosomes, Carboplatin, Drug resistance, Canine

## Abstract

**Background:**

Osteosarcoma patients often experience poor outcomes despite chemotherapy treatment, likely due in part to various mechanisms of tumor cell innate and/or acquired drug resistance. Exosomes, microvesicles secreted by cells, have been shown to play a role in drug resistance, but a comprehensive protein signature relating to osteosarcoma carboplatin resistance has not been fully characterized.

**Methods:**

In this study, cell lysates and exosomes from two derivatives (HMPOS-2.5R and HMPOS-10R) of the HMPOS osteosarcoma cell line generated by repeated carboplatin treatment and recovery, were characterized proteomically by mass spectrometry. Protein cargos of circulating serum exosomes from dogs with naturally occurring osteosarcoma, were also assessed by mass spectrometry, to identify biomarkers that discriminate between good and poor responders to carboplatin therapy.

**Results:**

Both cell lysates and exosomes exhibited distinct protein signatures related to drug resistance. Furthermore, exosomes from the resistant HMPOS-2.5R cell line were found to transfer drug resistance to drug-sensitive HMPOS cells. The comparison of serum exosomes from dogs with a favorable disease-free interval [DFI] of > 300 days, and dogs with < 100 days DFI revealed a proteomic signature that could discriminate between the two cohorts with high accuracy. Furthermore, when the patient’s exosomes were compared to exosomes isolated from carboplatin resistant cell lines, several putative biomarkers were found to be shared.

**Conclusions:**

The findings of this study highlight the significance of exosomes in the potential transfer of drug resistance, and the discovery of novel biomarkers for the development of liquid biopsies to better guide personalized chemotherapy treatment.

## Introduction

Osteosarcoma is the most common form of bone cancer with an increased incidence in adolescents and young adults [1]. Risk factors include Paget disease, osteitis deformans, ionizing radiation, inherited genetic conditions (such as Li-Fraumeni syndrome), and mutation of the retinoblastoma (*RB*) gene [2–4]. Most cases exhibit a primary lesion in the appendicular skeleton on presentation and despite surgical excision and chemotherapy treatments, the disease can metastasize to the lungs, which is the leading cause of death in these patients [1, 2].

Canine osteosarcoma disease shares many characteristics with the disease in human, including etiology, location in the appendicular skeleton, histology, tendency to metastasize to the lung, and overall metastatic rate [5–7]. However, the incidence rate of osteosarcoma is much greater for dogs than humans. In combination with the inherently shorter lifespan of dogs, the manifestation of spontaneous disease and the multifactorial etiology, allows for enhanced data collection and the development of a clinically relevant model [6]. Like humans, surgical excision of the primary disease, followed by a chemotherapy regimen, have been the standard of care for dogs; however, despite multimodal therapies, prognosis remains poor [6, 8].

Platinum chemotherapy agents have been used as the mainstay treatment of osteosarcoma in both species, either as part of a multidrug protocol or as a single agent [2, 8]. The mechanisms of resistance to carboplatin and cisplatin are similar despite differences in chemical structure, pharmacokinetics, pharmacodynamics and toxicity [9]. Malignant cells can exhibit intrinsic or acquired resistance to these drugs during treatment [9]. Documented resistance mechanisms include, reduced drug accumulation, inactivation by metallothioneins or glutathione (GSH), resisting apoptosis, potentiation of the cell cycle, and increased DNA repair [10]. While genetic and epigenetic changes in malignant cells primarily drive drug resistance, recent studies highlight other factors, such as the tumor microenvironment and microvesicles, including exosomes, as significant contributors in these processes [10–13].

Exosome vesicles, which are 30–150 µm in diameter, contain DNA, RNA, proteins and lipids, are secreted by all cells [14, 15]. The proteomic signature of exosomes can reflect originating cell type and physiological status [15]. These unique signatures can be used for the discovery of novel biomarkers and the design of liquid biopsies in diseases like cancer [16]. Malignant cell exosomes are capable of autocrine and paracrine signaling to promote tumor progression by shifting cellular metabolism, increasing angiogenesis, or advancement of immune evasion [17–19]. Interestingly, several studies have demonstrated exosomal transference of platinum agent resistance to sensitive cells that ultimately increases the probability for treatment failure [13, 20, 21].

The goal of the current study was to investigate canine osteosarcoma as a spontaneous model to explore the role exosomes play in mechanisms of carboplatin resistance and the utility of exosomal proteins as prognostic biomarkers for this disease. Carboplatin-resistant canine osteosarcoma cell lines were generated, and secreted exosomes were characterized proteomically. Exosomes from these resistant cells effectively transferred drug-resistance to sensitive cells and were shown to exhibit a unique cargo that correlate with their resistance profile. Serum exosomes were also isolated from canine osteosarcoma patients with both, good and poor responses to carboplatin-based chemotherapy (favorable disease-free intervals [DFI] (> 300 days), and poor responders [< 100 days]). The proteomic signature of serum-isolated exosomes from dogs with spontaneous osteosarcoma disease, could discriminate between the dog with favorable prognosis in comparison to the poor responders with high specificity. These data demonstrate the utility of exosomes as a significant source of biomarkers for the design of precision medicine, and their potential role in horizontal transition of platinum resistance between malignant cells.

## Materials and methods

### Cell lines

HMPOS cells were a kind gift from the Barroga and Fujinaga lab [22]. The original and derived cells, were tested for Mycoplasma prior, and during the study. To confirm the authenticity of the cells, an alkaline phosphatase assay was performed on all cell lines.

### Generation of carboplatin resistant cell lines

The HMPOS cells were grown in media (RPMI 1640 and 10% FBS supplemented with 100 μg/mL Streptomycin and 100 units/mL Penicillin) and incubated at 37 °C with 5% CO_2_ [22]. The cells were treated with gradually increasing concentrations of carboplatin starting at 0.5 μM. Following a 72 h incubation period, surviving cells were subsequently expanded and passaged at least once before the next treatment. If cells were at less than 70% confluency after 72 h of treatment, the same dose was administered again for another 72 h. If cells were at 70% confluency or greater, then the dose was increased one increment until a concentration of 10 μM was reached. The carboplatin-resistant HMPOS cells isolated at 0, 2.5 and 10 uM are referred to as HMPOS-S, HMPOS-2.5R, and HMPOS-10R, respectively.

### Carboplatin proliferation assay (MTS)

HMPOS cell lines were grown to confluence. Next, 10,000 cells from each cell line were plated onto a 96 well plate in triplicate in 200 μL of media. Cells were then incubated for 24 h and subsequently serum starved for an additional 24 h. The cells were then treated with media (vehicle control), 0.5, 1, 2.5, 4, 6, 8, and 10 μM carboplatin diluted in RPMI media for 72-h. Next, 130 μL of the cell culture media from each well was collected and 50 μL of a mixture of RPMI 1640 + CellTiter 96™ AQueous One Solution Cell Proliferation Assay reagent, prepared per manufacturer’s instructions (Promega), was added to each well. Cells were incubated for 4 h at 37 °C. Wells were measured at 490 nm and the absorption was normalized to the control treatment.

### Determination of generation time

HMPOS-S, -2.5R, and -10R cells were plated in 6-well plates at a density of 100,000 cells per well in triplicate. After a 48-h incubation period cells were counted using a hemocytometer. The generation time of these cells was calculated according to the following formula::$t=H\mathit{ln}2/\mathit{ln}\left({c}_{2}/{c}_{1}\right)$, where *H* = time elapsed between plating and counting, *c*_*2*_ = count of cells 48 h after plating, and *c*_*1*_ = count of cells upon plating (100,000) [23]. The generation time values for each triplicate were averaged together and repeated for a total of three independent replicates.

### Cell cycle phase analysis

Isolated cells were fixed in 70% EtOH overnight then 25 μL of RNase A (Thermo Scientific) and 10 μL of propidium iodide (final concentration 0.6 μg/mL) were added. Cells were interrogated using a Cytoflex FACS (BD Coulter). Software analysis was performed using the CytExpert software (BD Coulter).

### Exosome isolation, quantification, and validation

HMPOS cell lines were grown as previously described [17]. Once cells reached 60–70% confluency, their media was changed to contain RPMI 1640, 100 μg/mL Streptomycin and 100 units/mL Penicillin, and 10% exosome depleted FBS (ThermoFisher) at a total volume of 15 mLs. Cells were grown for 24–48 h, after which the media was collected and stored at − 20 °C. Pooled media was thawed at 37 °C and spun at 10,000 rpm for 30 min on a high-speed centrifuge (Eppendorf 5804R, rotor F34-6-38). The supernatant was collected and transferred into 10.4 mL ultracentrifuge tubes (Beckman Coulter) and centrifuged at 40,000 rpm for 70 min (Beckman Coulter Optima XE-90 Ultracentrifuge, rotor type 70.1 Ti, fixed angle). After removing the supernatant, the exosome pellet was re-suspended in PBS and sterile filtered through a 0.22 μM filter (Millex-GV filter, 4 mm diameter, SLGV004SL). Exosome suspensions were aliquoted and diluted by a factor of 2.5 for quantification. These dilutions were quantified relative to their protein content.

Exosomes were characterized using the NanoSight NS500 instrument (NanoSight Ltd., Amesbury, United Kingdom, NTA version 3.0 0064). Light scattered by the particles under Brownian motion were captured for 60 s. Samples were diluted to obtain the concentration between 50 and 150 particles per frame. Each sample was injected into the chamber (temperature: 25 °C, viscosity of PBS 0.912–0.913 cP) and the setting of the system were adjusted as follows: camera level: 16, slider shutter: 1300, slider gain: 512, Frames Processed: 851, Frames per Second: 14.2, Blur size: Auto; Detection Threshold: 5). The three videos for each sample were analyzed to obtain the mean, mode, and number of particles per milliliter.

### Exosomal modulation of carboplatin resistance

Carboplatin-sensitive HMPOS-S cells (10,000) were plated into a 96 well plate in triplicate and incubated for 24 h at 37 °C. To synchronize the cells, cultures were incubated for 24 h in FBS free media. Cells were then pre-treated with 10 μg of HMPOS-S isolated exosomes, HMPOS-2.5R isolated exosomes, HMPOS-10R isolated exosomes, or a PBS vehicle control for an additional 24 h. Carboplatin was then added to the wells at concentrations of 0, 2.5, and 10 μM and cells were incubated for 72 h. At the 24 h and 48 h timepoints, relative to when carboplatin was first added, fresh exosomes and carboplatin were added as described above. After 72 h, cells were analyzed with the MTS reagent as previously described.

### Mass spectrometry

Cell lysates from HMPOS cell lines were prepared following 10 cycles of rapid freeze/thaw. The crude lysate was purified using chloroform–methanol. Exosomes were isolated and quantified as previously described. A total of 50 μg of cell lysate and exosomes (relative to protein concentration) were taken to the OSU Mass Spectrometry Center for analysis. The proteins were digested by sequencing grade modified trypsin.

Peptide analysis was achieved using an Orbitrap Fusion Lumos mass spectrometer with a Nano ESI source (Thermo Scientific) coupled with a Waters nanoAcquity UPLC system (Waters, Milford, MA). The proteolytic products were desalted and loaded on a nanoAcquity UPLC 2 G Trap Column (180 μm × 20 mm, 5 μm) for 5 min with solvent 0.1% formic acid in 3% ACN at a flow rate of 5 μL/min. A nanoAcquity UPLC RPeptide BEH C18 column (100 μm × 100 mm, 1.7 μm) was applied to separate peptides following by a 120-min gradient consisting of 0.1% formic acid in H2O (mobile phase A) and 0.1% formic acid in ACN (mobile phase B), where B was increased from 3 to 10% at 3 min, 10% ® 30% at 105 min, 30% ® 90% at 108 min and held 4 min, and then decreased to 3% at 113 min and held until 120 min. The LC flow rate was set at 500 nL/min. All mass spectral data were acquired in the positive ion mode. The spray voltage was 2400 V and the ion transfer tube temperature was 300 °C. MS and MS/MS spectra were acquired by the Orbitrap analyzer (resolution 120 K at m/z 200) and Ion Trap (collision induced dissociation CID) respectively. Automatic gain control target was set to 4.0 × 10^5^ for precursor ions and 104 for product ions. Mass tolerances were set at ± 10 ppm for precursor ions and 0.6 Da for-fragment ions.

Raw data were analyzed with Thermo Scientific Proteome Discoverer 2.2 software and searched initially against the Uniprot Canis database using Sequest HT as search engine. A maximum of two missed cleavage sites was allowed. Carbamidomethylation of cysteine and oxidation of methionine were specified as static modification and dynamic modification, respectively. The overall false discovery rate (FDR) at the protein level was less than 1%. To allow for GO annotation analysis, the datasets were also searched against the Uniprot Homo sapiens protein database. Canine and human database search results were submitted to a Basic Local Alignment Search Tool (Blastp) analysis in Uniprot website (https://www.uniprot.org/blast/). Only canine proteins with more than 99% amino acid sequence similarities with its human ortholog were retained.

The fold change (FC) of a given protein was defined as the ratio of abundance between two groups (ex. HMPOS-S vs HMPOS-10R). To calculate the FC of a given protein, each peptide group ratio was first calculated as the geometric median of all combinations of ratios from all the replicates in the same group. Secondly, the protein ratio was subsequently calculated as the geometric median of the peptide group ratios. Overall, the ratio for protein X reflects the ratio of abundance of protein X in HMPOS-S controls, relative to the abundance of protein X in other samples. The protein FC between the following groups was investigated: (1) HMPOS-S vs HMPOS-2.5R vs HMPOS-10 R cell lysate and (2) HMPOS-S vs HMPOS-2.5R vs HMPOS-10R exosomes.

### Immunoblotting

Osteosarcoma HMPOS cells were isolated and lysed in RIPA buffer (Bio-Rad). Quantified lysate protein (10 ug) was mixed with LDS Sample Buffer (Invitrogen) and loaded on to pre-cast 4–12% Bis–Tris gels. Electrophoresis of gels was at 100 V for 1–2 h (Life Technologies, Eugene, OR). Proteins were transferred to nitrocellulose membranes and membranes blocked for 1 h in Blocking Buffer (LI-COR, Lincoln, NE). Membranes were then incubated with the following: rabbit anti-canine non-phosphorylated (Active) β-catenin (Ser45) (Cell Signaling Technologies), rabbit anti-canine phospho-β-catenin (Ser33/37/Thr41) (Cell Signaling Technologies), and β-Actin Mouse Antibody (Santa Cruz Biotechnologies). After washing with buffer (TBS, 10% Tween), membranes were incubated with the following secondary antibodies: goat anti-mouse IgG-HRP (Santa Cruz Biotechnologies), and goat anti-rabbit IgG-HRP (Santa Cruz Biotechnologies). Membranes were developed using the Supersignal West Femto Maximum Sensitivity Chemiluminescent Substrate (Thermo Fisher).

### Patient sera collection

Patient sera was obtained from historical samples stored at the Oregon State University Biorepository. All samples collected to the biorepository at Oregon State University. The Oregon State University Institutional Animal Care and Use Committee (IACUC) exempted this study from full review. Serum was taken prior to therapy from adult (> 1 year) medium- and large-breed dogs (> 20 kg) with pathology-verified appendicular osteosarcoma. All Patients underwent standard of care treatment which included an amputation of the affected limb, and carboplatin chemotherapy (270–300 mg/m^2^ administered every 3 weeks for a total of 4 doses).

### Statistical analyses

All statistical analysis was performed on GraphPad Prism Software. MTS assay validation of carboplatin resistance, generation time data, and luminometry data, were all analyzed using a one-way ANOVA analysis, followed by Dunnett’s Multiple Comparisons test to analyze significance relative to appropriate controls. MTS assay data from treatment of sensitive HMPOS cells with carboplatin and exosomes was subjected to a two-way ANOVA analysis, followed by Bonferroni post-tests to compare all potential significant interactions. Statistical significance was assigned based on p < 0.05 (*), p < 0.01 (**), and p < 0.001 (***).

To generate graphical representations of the canine patient serum exosome data, protein abundance values were normalized to the sum of each column, and all values were subjected to a t-test. The heatmap rows are ordered by t-score between the sample groups. Red coloration represents higher expression relative to the average of the first five columns (the patient cohort that responded well to chemotherapy), and blue represents lowered expression.

### Results

### Characterization of carboplatin resistant HMPOS osteosarcoma cell lines

**Fig. 1:**
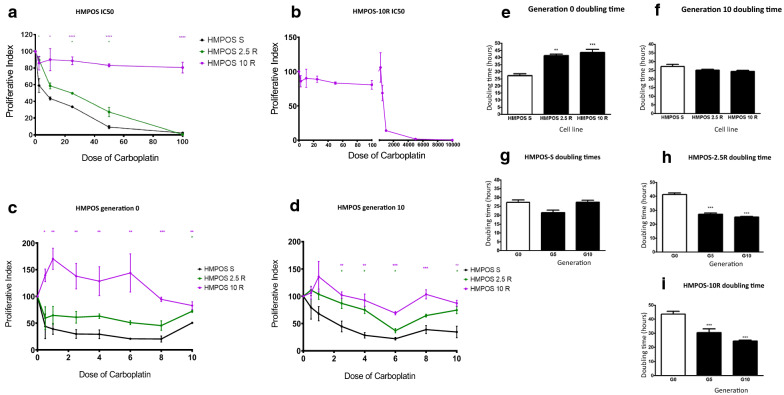
**a** Half maximal inhibitory concentration (IC50) demonstrates the increased resistance to carboplatin from the sensitive (HMPOS-S) to the most resistant cells (HMPOS-2.5R and HMPOS-10R), the IC 50 of HMPOS-10R is plotted in **b** (n = 3).** b** HMPOS 10 R IC50 exhibit a substantially high value in comparison to the other cell lines (n = 3). **c** Cell viability assay of generation 0 demonstrates a significant resistance to carboplatin at all concentrations for the HMPOS-10R cell lines. The HMPOS-2.5R cells exhibit resistance only at the highest concentration of the drug (n = 3, [*] = P < 0.05, [**] = P < 0.01, [***] = P < 0.001). **d** Sensitive and resistant HMPOS, Generation 10 cell lines. **e** Doubling time of HMPOS generation 0, sensitive and resistant cell lines (n = 3, [**] = P < 0.01, [***] = P < 0.001). A significant increase in doubling time was evident in HMPOS-2.5R (P < 0.01, [**]) and HMPOS-10R (P < 0.001, [***]) (n = 3) **f** Doubling time for HMPOS-S and resistant cell lines of generation 10 demonstrate that HMPOS-2.5 and 10R returned to the original doubling time (n = 3). **g** Doubling time between three generations (0, 5 and 10) of HMPOS-S show no statistically significant difference (n = 3). **h** Comparison of doubling time of HMPOS-2.5R through the examined generations (0,5,10) is significantly decreased with the later generations (n = 3, [***] = P < 0.001).**i** Cross generational comparison for the doubling time of HMPOS-10R also demonstrate a significant decrease in generations 5 and 10 (n = 3, [***] = P < 0.001)

Two carboplatin-resistant derivative cell lines HMPOS-2.5R and HMPOS-10R were chosen for further study. After the initial derivation of these cell lines, carboplatin resistance was validated by the determination of the IC 50 of these cells in the presence of increasing concentrations of the drug (Fig. 1a and b). In comparison to the parental cells (HMPOS-S), HMPOS-10R cells were significantly more resistant at all concentrations, while HMPOS-2.5R cells exhibit significant drug resistance in higher concentrations. To quantify the extent of drug resistance, each cell line was treated with a continually increasing concentration of carboplatin. The IC50s for HMPOS-S, HMPOS-2.5R and HMPOS-10R cells were estimated to be 5.62 μM 18.2 μM and 664.2 μM of carboplatin, respectively.

The doubling time of the parental and the derived resistant lines was calculated by cell plating and subsequent counting and mathematical analysis. For generation 0, HMPOS-S cells doubled every 27.2 h, while HMPOS 2.5-R cells doubled every 41.28 h, and HMPOS-10R cells doubled every 43.52 h (Fig. 1f). Based on these times cells were then (i) passaged for five generations in the absence of carboplatin, (ii) assessed for doubling time, (iii) passaged for five more generations, and (iv) a final assessment of doubling time and carboplatin resistance was initiated after the tenth generation. Tenth generation HMPOS- 10R cells exhibited similar carboplatin resistant when compared to their own first-generation cells and when compared to parental HMPOS-S (Fig. 1c, d). The HMPOS-2.5R cells have shown an even more robust resistance at generation 10 in comparison to the early generations. The doubling times of tenth generation HMPOS-2.5R and -10R cells were identical to tenth generation sensitive (HMPOS-S) cells but significantly shorter than first generation resistant cells (Fig. 1e–i).

### Proteomic analysis of carboplatin resistant HMPOS cells and exosomes

**Fig. 2:**
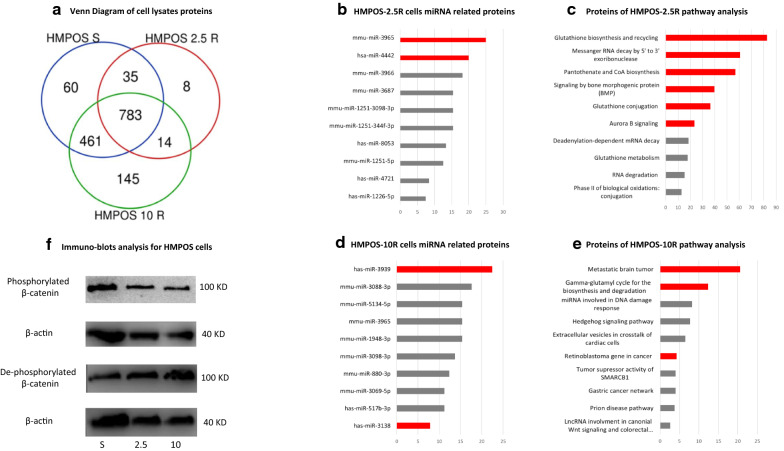
**a** Venn diagram of the proteins extracted from cell lysates of HMPOS-S, HMPOS-2.5R and HMPOS-10R show that majority of proteins are shared between the three cell lines. HMPOS-S express 60 unique proteins while HMPOS-2.5R and HMPOS-10R express 8 and 145 unique proteins respectively. **b** HMPOS-2.5R cell lysates miRNA associated proteins. Columns represent odds ratio values; the red colored columns correlate to P value of < 0.05. **c** HMPOS-2.5R cell lysates pathway analysis of expressed proteins (columns in red have P value of < 0.05). The glutathione biosynthesis and recycling and conjugation pathways exhibit a significant P value which is in line with their resistance to carboplatin. **d** HMPOS-10R cell lysates miRNA. **e** HMPOS-10R cell lysates pathway analysis exhibit significance of pathways associated with platinum agents drug resistance such as gamma-glutamyltrasferase synthesis. **f** Immunoblot assay for HMPOS-S, HMPOS-2.5R and HMPOS-10R cell lysates, exhibit an increased concentration of de-phosphorylated β-catenin with increased cellular resistance to carboplatin

Cellular proteins from HMPOS-S, -2.5R, and -10R cells were analyzed by mass spectrometry. Following peptide analysis of the three cell lines, a total of 145 unique peptides were measured from HMPOS-10R cell lysates and 8 unique peptides were measured from HMPOS-2.5R that were differentially expressed compared to parental HMPOS-S (Fig. 2a) (Table 1). After comprehensive gene set enrichment, proteins from HMPOS-2.5R and HMPOS-10R cell lysates that were 100-fold more abundant in comparison to HMPOS-S cells were analyzed in relation to cellular pathways and miRNA-protein interactions (Fig. 2b–e) [24]. In HMPOS-2.5R cells, a significant increase in proteins associated with glutathione synthesis, recycling, and conjugation pathway alterations was observed, while HMPOS-10R cells exhibited altered pRB activity and abnormal DNA methylation.

**Table 1:** Selected over expressed proteins in cell lysates of carboplatin resistant cell lines

Protein name	2.5 R	10 R	Biological function
Gamma-glutamylcyclotransferase	✓		Glutathione Metabolism, EMT regulation via PI3K/AKT/mTOR
Heat shock Protein 27		✓	Anti-apoptotic
S100A7	✓	✓	EMT, migration, invasion
Cyclin-dependent kinase 6		✓	Cell Cycle Progression
histone deacetylase		✓	Chromatin Remodeling
14-3-3 protein sigma		✓	Cell Signaling
Cyclin-dependent kinase 2		✓	Cell Cycle Progression
Insulin-like growth factor 2 mRNA-binding protein 3		✓	Stabilizing IGF-2 mRNA
β-catenin	✓	✓	Cell Signaling
Transgelin		✓	Stemness, invasion
Follistatin-related protein 1	✓	✓	Marker of EMT, invasion
Stathmin		✓	Cell cycle arrest via microtubule depolymerization
14-3-3 protein theta		✓	Cell Signaling
Insulin-like growth factor 2 mRNA-binding protein 2		✓	Stabilizing IGF-2 mRNA
Sialidase Neu2		✓	Invasion, metastasis, differentiation state
Glutamine synthetase		✓	Glutamine metabolism
Alpha-2-macroglobulin	✓	✓	Protease inhibitor, stemness, platinum inactivation
Adenosylhomocysteinase		✓	Alteration of DNA methylation via Adenosylhomocysteine level control
UV excision repair protein RAD23 homolog A		✓	DNA repair
N-Cadherin	✓	✓	Marker of EMT
Secreted frizzled-related protein 2		✓	Wnt signaling, tissue regeneration
Gamma-enolase		✓	Increased glycolysis

**Fig. 3:**
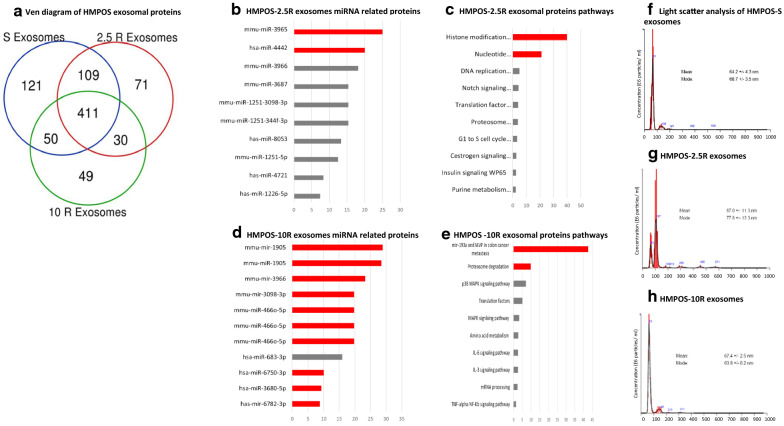
**a** Venn diagram identify 411 shared proteins the exosomes extracted from HMPOS-S, HMPOS-2.5R, and HMPOS-10R, and 121, 71 and 49 unique proteins respectively. **b** HMPOS-2.5R exosomal miRNA associated proteins, the colored columns correlate to P value of < 0.05. **c** HMPOS-2.5R Exosomal proteins analysis revealed the upregulation of histone modification and nucleotide metabolism pathways which correlate with platinum agents drug resistance.** d** HMPOS-10R display exosomal miRNA related proteins (columns in red have P value of < 0.05). **e** HMPOS-10R exosomal protein analysis. **f** light scatter analysis of exosomes extracted from HMPOS-S cells supernatants exhibit nanoparticle size with a mean of 64.2 nm. **g** Light scatter analysis of exosomes extracted from HMPOS-2.5R cells supernatants with a mean value of 87 nm. **h** light scatter analysis of exosomes extracted from HMPOS-10R cells supernatants exhibit a mean value of 67.4 nm

Proteins collected from enriched exosomes secreted by HMPOS-S, -2.5R, and -10R cells were also analyzed by mass spectrometry. Proteins extracted from the exosomes of the three cell lines exhibit a distinct cargo signature for each one of them (Fig. 3a)(Table 2). A pathway analysis was performed on the most abundant proteins in HMPOS-2.5R and -10R exosomes and HMPOS-S (Fig. 3b–e). In addition, we have performed an analysis of miRNA predicted to interact with the proteins identified for the three cell lines. Proteins involved in nucleotide metabolism and histone modification were highly represented in HMPOS-2.5R exosomes, and proteins representative of proteosomal degradation and miR-193a signaling were more abundant in exosomes secreted by HMPOS-10R. The miRNA predicted to interact with the proteins of the HMPOS-2.5R and HMPOS-10R relate to characteristic of stemness, resistance and self-renewal (e.g. miR-4721 and miR-760). Selected exosomal proteins of interest found to be abundant in resistant HMPOS-2.5R and HMPOS-10R compared to HMPOS-S are provided in Table 2.

**Table 2:** Selected Overexpressed proteins in exosomal cargo of carboplatin resistant cells

Protein Name	2.5 R	10 R	Biological Function
Metallothionein-1G	✓	✓	Carboplatin Inactivation
Heat shock Protein 105 kDa		✓	β-catenin dephosphorylation via protein phosphatase 2A recruitment
S100A10	✓	✓	Conditioning distant sites for metastasis via exosomes
LDLR chaperone MESD		✓	Wnt Signaling
Integrin beta	✓	✓	Conditioning distant sites for metastasis via exosomes
14–3-3 protein eta		✓	Cell Signaling
ILF3		✓	Promotes Survivin expression
Insulin-like growth factor 2 mRNA-binding protein 1		✓	Stabilizing IGF-2 mRNA
β-catenin	✓	✓	Cell Signaling
Transgelin	✓	✓	Stemness, invasion
Heat shock Protein 27		✓	Anti-apoptotic
Follistatin-related protein 1	✓	✓	Invasion
Stathmin	✓	✓	Cell cycle arrest via microtubule depolymerization
Heterogeneous nuclear ribonucleoprotein Q	✓	✓	miRNA loading into exosomes
Histone H1.3	✓	✓	Binding of cis-platinated DNA
14–3-3 protein theta	✓		Cell Signaling
Gamma-enolase	✓		Glycolysis
WD repeat and HMG-box DNA-binding protein 1	✓		DNA Damage Response
SWI/SNF complex subunit SMARCC2	✓		Chromatin Remodeling
Serine hydroxymethyltransferase, mitochondrial	✓		Chromatin Remodeling
Histone deacetylase 2	✓		Chromatin Remodeling
Hypoxanthine–guanine phosphoribosyltransferase	✓		Nucleotide Metabolism
ribose-phosphate pyrophosphokinase 1	✓		Nucleotide Metabolism

### Exosomal effect on cell cycle phase

**Fig. 4:**
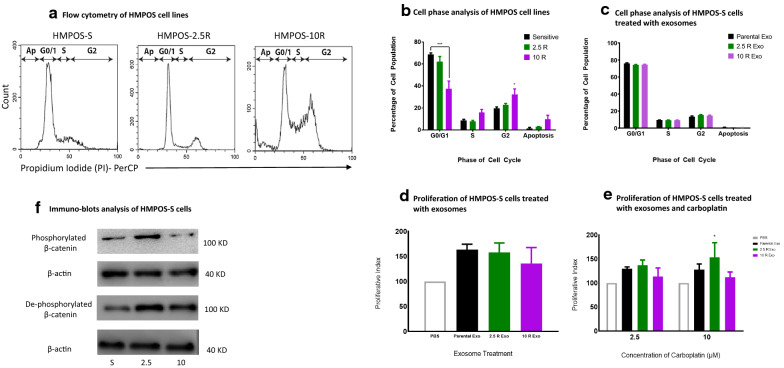
**a** Propidium iodide histogram of sensitive and resistant HMPOS cell lines demonstrate the accumulation of HMPOS-10R at the G2/M phase. **b** Cell Phase of sensitive and resistant cell lines exhibit a significant decrease of the HMPOS 10 R cells in the G0/G1 phase and their accumulation at the G2/M phase (n = 3, [*] = P < 0.05, [***] = P < 0.001). **c** Exosomes extracted from sensitive and resistant cells lines coincubation with HMPOS S cells show no changes in cell phase (n = 3). **d** HMPOS-S Co-incubation with Exosomes of sensitive and resistant cell lines demonstrate an increased proliferation independent of the exosome’s origin (n = 3). **e** HMPOS-S Co-incubation with Exosomes of sensitive and resistant cell lines and with carboplatin demonstrating increased resistance of these cells to high concentration of carboplatin following coincubation with exosomes from HMPOS-2.5R (n = 3, [*] = P < 0.05). **f** Immunoblot assay for HMPOS-S cells demonstrate the decrease of phosphorylated β-catenin and increased of dephosphorylation following coincubation with exosomes extracted from supernatants of carboplatin resistant cell lines

Flow cytometry analysis showed that majority of the HMPOS-S and -2.5R cells accumulated at the G0/1 cell phase, while the HMPOS-10R cells exhibited a significant accumulation at the G2 cell phase (Fig. 4a and b). Flow cytometry of HMPOS-S cells that were treated for 24 h with resistant cell exosomes did not produce any significant differences of cell accumulation at the different cell cycle phases (Fig. 4c).

### Exosomal transfer of carboplatin resistance to sensitive cells

To elucidate the effects that exosomes derived from HMPOS-2.5R and HMPOS-10R cells may have in the transfer of chemotherapy resistance, sensitive HMPOS-S cells were cultured with exosomes extracted from the drug resistant cells. While the addition of exosomes did not modify the proliferation of HMPOS-S cells in comparison to untreated cells, the combination of exosomes from HMPOS-2.5R and carboplatin exhibit a statistically significant difference in cell viability was seen at the higher 10 μM dose (Fig. 4d and e). To examine the modulatory effects of β-catenin activation, resistant exosome-treated HMPOS-S cell lysates were probed for phosphorylated β-catenin and immunoblotting revealed a decrease in phosphorylated β-catenin following incubation with HMPOS-10R exosomes (Fig. 4f). Interestingly, protein levels of phosphorylated β-catenin increased in HMPOS-S cells treated with HMPOS-2.5R exosomes. Measurement of dephosphorylated β-catenin demonstrated a notable difference in protein levels, as HMPOS-S cells treated with both HMPOS-2.5R and HMPOS-10R exosomes showed increases in dephosphorylated β-catenin levels, with HMPOS-2.5R exosomes exhibiting the largest increase (Fig. 4f).

### Proteomic analysis of canine osteosarcoma patient serum exosomes

**Fig. 5:**
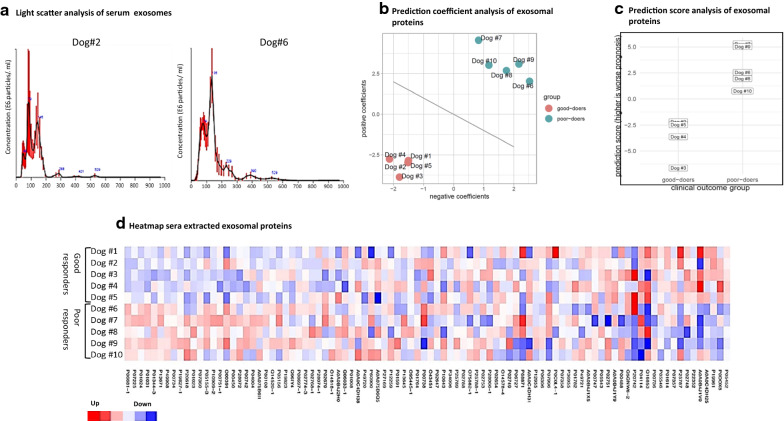
**a **Light scatter analysis of exosomes extracted from the serum of a dog from the “good doer” cohort (dog #2), and dog from the “poor doer” cohort (dog #6) demonstrate the extracted exosome size and concentration. **b** Each mark represents a serum exosome sample, color-coded by outcome. Horizontal axis represents the net prediction score contribution from serum exosome protein features that are negatively correlated with good outcome, for that sample. Vertical axis represents the net prediction score contribution from serum exosome protein features that are positively correlated with good outcome, for that sample. Line denotes the decision boundary for predicting outcome. **c** Each mark represents a serum exosome sample, grouped by outcome. Vertical axis represents the net prognosis prediction score (lower score means better prognosis; higher score means worse prognosis). **d** Each cell in the heatmap represents a combination of serum exosome sample (row) and protein identified by UniProt accession (column). Heatmap color indicates relative log2 expression of the indicated protein in the specified sample relative to the column average (see Methods). Heatmap of the protein expression demonstrate distinct patterns between the cohort of good doers (Dog #1–5) and poor doers (Dog #6–10)

Exosomes were isolated from the serum of ten patients, and two representative samples of each cohort were validated by NanoSight analysis (Fig. 5a). Patients were grouped into two cohorts (n = 5 per cohort) based on responsiveness to amputation and adjuvant carboplatin chemotherapy. Good responders were defined by a disease-free interval (DFI) of > 300 days and poor responders defined by a DFI of < 100 days (Table 3). The respective proteomes of each patient’s serum exosomes were probed using mass spectrometry. A preliminary analysis of each cohort was created for all identified peptides after statistical testing and normalization revealing a significant correlation between the clustering of the exosomal proteins and patient outcome (Fig. 5b) (Table 4). Prediction score analysis revealed a distinct discrimination between the exosomal proteins of patients with good and bad outcomes (Fig. 5c).

**Table 3:** Participating dogs demographic, treatments and survival data

Patient	Breed	Age	Sex	Location	Treatment	DFI (days)	OST (days)
1	Great Dane	9	SF	L distal radius	Sx/Carboplatin	340	420
2	Saint Bernard	6	SF	R distal radius	Sx/Carboplatin		
3	Doberman	5	SF	L distal radius	Sx/Carboplatin	308	316
4	Pitbull Mix	7	NM	L distal radius	Sx/Carboplatin	688 <	688 <
5	German Shepherd	7	SF	R distal radius	Sx/Carboplatin	676	783
6	Saint Bernard	5	NM	L distal radius	Sx/Carboplatin and doxorubicin (rescue)	58	81
7	Newfoundland	5	SF	R distal radius	Sx/Carboplatin and doxorubicin (rescue)	70	165
8	Doberman	7	NM	L distal radius	Sx/Carboplatin,	62	196
9	Mixed	10	NM	R distal radius	Sx/Carboplatin and doxorubicin, Dasatinib (rescue)	88	372
10	Golden Retriever	9	NM	R proximal humerus	Sx/Carboplatin and doxorubicin, listeria vaccine (rescue)	61	131

SF: Spayed female; NM: neutered male; Sx: surgery (amputation); DFI: disease free interval; OST: Overall survival time

**Table 4:** Discriminating proteins isolated from circulating exosomes

Protein name	Good responder	Poor responder	Biological function
Tetranectin	✓		Plasminogen-binding protein, positive prognostic indicator in several cancers
Complement C2		✓	Complement response, poor prognostic indicator in multiple cancers
Alpha-2-macroglobulin		✓	Protease inhibitor, poor prognostic indicator in multiple cancers
Protein S		✓	Coagulation protein, Poor prognostic indicator

## Discussion

Drug resistance of primary and metastatic disease is the main cause for treatment failure and ultimately patient mortality in many cancer types. There are immense efforts to identify the mechanisms associated with chemotherapy resistance, to improve treatment outcomes, and reduce adverse effects associated with these treatments. Recent studies revealed the critical role of exosomes in chemotherapy resistance, which was linked to their microRNA cargo; however, the role of other exosomal components have not been thoroughly investigated [25, 26]. In the current study we have identified proteins associated with carboplatin drug resistance generated in vitro and demonstrated the acquisition of this resistance by sensitive cells following exposure to exosomes from drug-resistant cells. In vivo examination has identified several proteins in circulating exosomes that may have the potential to predict responses to carboplatin treatment in canine patients with osteosarcoma.

The resistant cell lines in this study evolved through repeated and increased challenge of HMPOS cell line. While all these cell lines originated from the same clone, they demonstrated vastly different profiles of drug resistance. The HMPOS-10 R line exhibits an IC50 which was more than 36-fold higher in comparison to the HMPOS-2.5 R. Interestingly, once adapted, the HMPOS-10 R cells have maintained their resistance for at least 10 generations even in the absence of constant treatment with carboplatin. In comparison, the HMPOS-2.5R cells exhibit a significant gain of resistance between the first generation and the 10th. These changes can be explained the gain of mutations of actively proliferating cells, or by epigenetic changes in the case of quiescent cells [27, 28]. It has been shown that through clonal selection and expansion, a stable resistance is achieved for clones with resistant mutations, or a more transient resistance in nature for cells resistance acquired via epigenetic changes [27, 29]. A future study of our model will be designed to investigate the nature of resistance acquisition of our cells and whether it is due to resistant mutations, epigenetic changes, or a combination of both.

Malignant cells can often exhibit an increased doubling time when exposed to cytotoxic drugs for the surveillance and repair of DNA damage [30]. We have documented a significant increase in the doubling time of HMPOS-2.5 R and -10 R on the first generations for these cell lines. However, the prolonged doubling time was eventually matched to the parent cell line (HMPOS-S) in generation 10 which is likely due to the acquisition of effective adaptation mechanisms. We can speculate that once the cells resolved the acute DNA damage induced by the increased concentration of carboplatin, new adaptation mechanisms were set. These acquired mechanisms ultimately allowed a more efficient carboplatin resistance which reduced DNA damage, allowing the cells to progress through their check points without delay.

Proteomic analysis of cell lysates highlighted the substantial differences between these lines based on their expression of discriminating proteins. Further analysis revealed the expression of Glutathione biosynthesis, recycling and conjugation pathway, and the Gamma glutamyl biosynthesis pathway in HMPOS-2.5R and HMPOS-10R cell line correspondently. These pathways promote the activation of glutathione S transferase (GST) enzyme that hydrolyze the active group of platinum agents and thus mitigate their effect on malignant cells [29, 31]. In addition, GST has a critical role in DNA repair, which ultimately promote evasion of apoptosis and cell survival [32, 33]. Drug resistance effect of proteins from the GST family was shown to be mediated via exosomes in breast cancer, but the significance of this mechanism in OS has not been investigated thus far [34].

The expression of β-catenin was upregulated in the carboplatin resistant cell lines, with higher abundance in the HMPOS-10R cells. β-catenin is an oncogene and a key protein in the canonical Wnt/ β-catenin signaling pathway [35]. It was shown to induce chemoresistance through multiple mechanisms, including upregulation of MDR1 and promotion of epithelial–mesenchymal transition [36, 37]. Immunoblot analysis of this protein revealed a distinct difference in the expression of phosphorylated vs dephosphorylated β-catenin in HMPOS-S, HMPOS-2.5R, and -10R cells (Fig. 2f), suggesting that β-catenin signaling was increased with drug resistance and may play a significant role in this process. When HMPOS-S cells were treated with exosomes derived from resistant cell lines, the expression of β-catenin was upregulated, and the larger portion of the total protein pool was dephosphorylated, confirming signal transduction of this pathway. Since the β-catenin protein was not detected in the exosomal cargo, it is possible that its expression was induced by the transfer of miRNA or other epigenetic factors. These preliminary findings suggest that β-catenin has a role in carboplatin drug resistance in osteosarcoma cell lines, and that exosomes of resistant cells may promote the expression and phosphorylation of β-catenin in naive cells. However, confirmation of the Wnt/β-catenin pathway activation was beyond the scope of this study and will require a confirmation of β-catenin nuclear translocation in future studies.

In spite the fact that exosomes from the HMPOS-10R induced the expression of β-catenin in HMPOS-S following coincubation it did not promote drug resistance in the latter line. This is in contrast with the induction of resistance following the coincubation of HMPOS-2.5R exosomes with HMPOS-S cells. It is surprising that the effect of exosomes on sensitive cells did not correlate with the degree of drug resistance of the donor cells, suggesting that this process is multifactorial and complex. The differences in the proteome and potentially miRNA cargoes of these exosomes are most likely the cause for these differences. For example, some proteins with protective effect such as the ribose-phosphate pyrophosphokinase 1 (PRPS1) which is responsible for nucleotide synthesis, and the ubiquitin ligase WD repeat and HMG-box DNA binding protein 1 (WDHD1) were expressed exclusively in the exosomal proteomic cargo of HMPOS-2.5R and may explain their distinct effect on HMPOS-S [38, 39]. While it is possible that the delivery and uptake of these proteins were key in promoting drug resistance, it is remained to be determined how other factors contained in the exosomal unit such miRNA might also support this phenomenon.

To identify reliable prognostic biomarkers, and validate our in-vitro model, we extracted exosomes from the blood sera of dogs with good and poor response to the standard of care. The extracted exosomes from both cohorts were subjected to proteomic analysis which revealed a cluster of biomarkers that could predict prognosis with 100% accuracy. Interestingly, there was only some overlap between the proteins expressed in exosomes from the resistant cell lines and the poor doer cohort. These findings are likely due to the low abundance of tumor origin exosomes and to tumors heterogenicity in vivo. In addition, exosomes in circulation derive from variety of tissues, and other than the malignant cells, represent cells from the the tumor stroma, and healthy tissues, which directly or indirectly might be impacted by tumor.

One of the proteins which has great promise as a potential biomarker is tetranectin. This protein was significantly reduced in carboplatin resistant cells in comparison to their parent cells (HMPOS-S). Tetranectin is plasminogen binding protein of the C-type family is expressed in the stroma of breast, pancreas, and gastric cancers [40–42]. It’s potential as a prognostic indicator was revealed when high levels in circulation of ovarian and colon cancer patients correlated with significantly better outcomes [43–45]. The notion of this protein as a biomarker for osteosarcoma was supported by the fact that the cohort of the poor doers in this study had a reduced expression of this protein, while the exosomes of good doers still expressed it. The consistencies between the in-vitro and in-vivo data support the model we have created and pave the way for future studies.

The role of the complement system in tumor progression has been documented in several cancers [46, 47]. Secretion of certain complement factors was linked to evasion of immune surveillance, mediation of epithelial to mesenchymal transition (EMT) and autocrine stimulation and overall decreased survival [47–49]. We have previously identified a significant correlation between the decrease of complement factor C1 and increased in its inhibitor (Serpin G1) in circulating exosomes of canine osteosarcoma patients [17]. In the current study however, we identified an increased expression of complement factor C2, in the poor doers’ cohort. While the source of this factor, its role in disease progression and drug resistance remains elusive, it should be considered as a potential biomarker and a therapeutic target.

We have also demonstrated the transition of drug resistance between two cell lines which was mediated by exosomes. While these results suggest a mechanistic description for drug resistance in tumors lacking innate resistance, it is important to note that this concept has been tested in-vitro and under very specific conditions. It is not clear how these factors would come to play if tested in-vivo and whether exosomes have the capacity to mediate drug resistance between clones of the primary and metastatic tumors. Another notable weakness of the study is the selection of canine patients that were used for the biomarker discovery. In the effort to make a clear distinction between the tested cohorts we harvested exosomes from patients that distinctively exhibited long, or short, disease-free intervals. To test the fitness of these biomarkers a larger, prospective study should be conducted in the future.

In conclusion, in this study we created carboplatin-resistant sublines of established canine osteosarcoma cell line HMPOS and characterized the proteome of these cells and their correlated exosomes. The exosomes extracted from the HMPOS-2.5 R cells exhibit a potential to mediate drug resistance in sensitive recipient cells. Exosomes extracted from the serum of osteosarcoma canine patients contain possible prognostic indicators such as: Tetranectin, as well as Complement C2 and C3 proteins. Strategies of carboplatin resistance potentiated by exosomes derived from carboplatin-resistant cells should be further validated as biomarkers and potential therapeutic targets for canine osteosarcoma.

## Data Availability

All data generated or analysed during this study are included in this published article as a supplementary materials.
